# Risk factors for elective and urgent open conversion after EVAR—a retrospective observational study

**DOI:** 10.1177/17085381221141118

**Published:** 2022-11-22

**Authors:** Haidar Haidar, Sebastian Kapahnke, Jan P Frese, Safwan Omran, Verena Mueller, Irene Hinterseher, Manuela Sommerfeld, Elena Kaschina, Frank Konietschke, Andreas Greiner, Matthias Buerger

**Affiliations:** 1 Department of Vascular Surgery, 14903 Charité — Universitätsmedizin Berlin, Corporate Member of Freie Universität Berlin, Humboldt-Universität zu Berlin and Berlin Institute of Health, Berlin, Germany; 2 Medizinische Hochschule Brandenburg Theodor Fontane, Neuruppin, Germany; 3 14903 Charité – Universitätsmedizin Berlin, Corporate Member of Freie Universität Berlin and Humboldt-Universität zu Berlin, Institute of Pharmacology, Center for Cardiovascular Research (CCR), Berlin, Germany; 4 14903 Charité – Universitätsmedizin Berlin, Corporate Member of Freie Universität Berlin, Humboldt Universität zu Berlin, Institute of Medical Biometrics and Clinical Epidemiology and Berlin Institute of Health (BIH), Berlin, Germany

**Keywords:** Open conversion, abdominal aortic aneurysm, endovascular aortic aneurysm repair, aortic wall, endoleak

## Abstract

**Background:**

Endovascular aortic aneurysm repair (EVAR) has become the standard procedure for treating infrarenal abdominal aortic aneurysms (AAA). Various associated complications can lead to open conversion (OC). Thorough follow-up after the procedure is mandatory for the early detection of complications. Persisting perfusion of the aneurysm, a so-called endoleak (EL), paired with structural instability because of aortic wall atrophy and impaired cell functionality induced by EVAR, results in a high risk for aortic rupture.

**Purpose:**

The goal of this study was to detect the risk factors for elective and urgent OC as a result of EVAR-induced pathophysiological changes inside the aortic wall.

**Research Design:**

Retrospective data analysis was performed on all open aortic repairs from January 2016 to December 2020.

**Data Collection and Analysis:**

Fifty patients were identified as treated by OC for failure of an infrarenal EVAR. The patients were divided into two subgroups, here depending on the urgency of surgery. Statistical analysis of patient characteristics and outcomes was performed.

**Results:**

The most common indications for OC were various types of EL (74%), resulting in an aortic rupture in 15 patients. Patients with insufficient or absent follow-up were treated more frequently in an emergency setting (16% vs. 63%). The mortality rate was higher in cases of emergency OC (3% vs. 26%).

**Conclusions:**

Particularly in cases of insufficient or absent follow-up, complications such as EL pose an enormous risk for fatal aortic rupture.

## Introduction

In recent decades, endovascular aortic aneurysm repair (EVAR)^1^ has become the standard procedure for treating infrarenal abdominal aortic aneurysms (AAA). Several prospective randomized clinical trials have demonstrated the superiority of EVAR when it comes to the elective treatment of infrarenal AAA compared with conventional open aortic repair (OAR), resulting in reduced mortality and morbidity rates in perioperative and short-term follow-up.^2–4^ However, this initial survival benefit is lost in the long-term follow-up, showing higher overall mortality and, in particular, higher aneurysm-related mortality after four to eight years.^5^

Furthermore, the need for secondary interventions after EVAR poses a further challenge.^4–6^ The most common indication for any reintervention after EVAR is the persistent perfusion of the aneurysm sac, which is called an endoleak (EL).^7,8^ If EL can be detected after EVAR, the risk of a fatal rupture increases.^5,9–19^ Other complications after EVAR include aortic graft infections (AGI),^20–22^ stent graft limb occlusions,^8^ or the formation of de-novo aneurysms in neighboring aortic segments.^23^ Although late rupture due to persisting EL or AGI seldom occurs after EVAR, the outcomes can be devastating.^24^

Although nearly 30 years passed after the first successful implantation of an endovascular stent graft, little is known about the underlying pathophysiological changes in aortic walls after EVAR leading to those complications. However, recently, increasing interest has developed on pathological alterations inside the aortic wall as a reaction to an inserted stent graft. In the case of EL, various theories^25–28^ suggest decreased structural and functional resistance to persistent blood flow inside the aneurysm. Structural atrophy and thinning of the aortic wall complemented by alterations in extracellular matrix components, such as collagen, may contribute to lower structural resistance.^25,28^ Impaired vascular smooth muscle contraction may support the decreased ability of the aortic wall to respond to newly occurring or persistent blood pressure inside the aneurysm sac after EVAR.^28^ In addition, we should consider the preconditions of an aneurysmatic changed aortic wall that we put our stent graft system inside. The pathology of aneurysm formations is based mainly on different genetic, epigenetic, and behavioral risk factors, leading to inflammation, oxidative stress, vascular smooth muscle cell apoptosis, and extracellular matrix degradation.^29,30^ Thus, aneurysmatic preconditions paired with pathological alterations induced by stent graft implantation may lead to severe complications. Once all interventional therapies have been exhausted, open conversion (OC) after EVAR may be the last resort for handling life-threatening complications, such as aortic rupture or fulminant AGI. Whereas some complications, such as asymptomatic sac growth induced by EL types II or IV, can be treated electively, particularly complications such as aortic rupture induced by EL types I or III, as well as fulminant AGI with accompanying gastrointestinal bleeding and septic shock, require immediate, life-saving surgery.

The goal of the current study was to investigate patient outcomes after elective and urgent OC as a result of various complications after EVAR. In particular, this explorative study examines the different indications resulting in elective or urgent OC as potential risk factors. We correlate our clinical outcome of patients treated by open conversion after EVAR to our previously published study, which provided evidence for impaired contractility in vascular smooth muscle cells and continuous degradation of extracellular matrix components, here resulting in aortic wall destabilization after EVAR.^28^ By analyzing the single-institution experience, valuable information about patients suffering from this comparably new and presumably increasing disease can be added to the literature and may contribute to an upcoming meta-analysis.

## Material and methods

The current study was approved by our institutional review board on 17 June 2020 (project identification code EA04/109/20). Because of the retrospective study design, there was no need for individual patient consent.

### Sample collection

Retrospective data analysis was performed for all OAR from January 2016 to December 2021. From patients’ records, we identified 50 patients treated by OC for an infrarenal EVAR failure.

Data on patient demographics, relevant comorbidities, the primary indication for EVAR, type of device, fixation site of the implanted device (supra-/infrarenal), participation in follow-ups, secondary interventions, operative details from the patients’ records, and clinical presentation at the time of OC were collected. We used information from preoperative computed tomography angiography (CT-A) to specify the exact indication for OC, including determining the type of EL. Moreover, we calculated the time between primary implantation and OC.

Procedural data included the time of surgery, surgical approach, clamp site (supra-celiac and infrarenal), in situ (ISR) or extra-anatomic reconstruction (EAR), the material used for re-construction (alloplastic and autologous), and completeness of graft removal (total, subtotal, and left in situ). The factors involved in choosing the surgical approach and extent of surgery included indication for OC, device, stent graft fixation site, and type of EL.

The outcomes evaluated were mortality (intraoperative, in-hospital, one-year, and overall mortality), complications requiring reintervention (e.g., acute limb ischemia (ALI), ischemic colitis, surgical site infection (SSI), and bleeding), acute kidney injury (AKI, defined as an absolute increase in serum creatinine ≥0.3 mg/dL (≥26.5 μmol/L) or ≥1.5- to 2.0-fold from baseline compared with initial serum-creatinine or decrease in urine production <0.5 mL/kg/h for six hours), myocardial infarction requiring catheter intervention, pneumonia (defined as typical symptoms supplemented by image morphological signs and microbiological diagnostic), and multiorgan failure (MOF, which is defined as a potentially reversible disorder affecting two or more organ systems).

The patients were divided depending on the urgency of surgery in elective OC (ELE-OC) and emergency OC (EM-OC). The decision was based on clinical presentation (hemorrhagic or septic shock and cardiopulmonary arrest) and image morphological findings (aortic rupture and aortointestinal fistula).

Regarding surgical treatment, all patients were evaluated and discussed for endovascular alternatives and, after decision was made for OC, the extent of stent graft removal. To reduce further long-time complications because of the mostly unknown underlying pathophysiological changes in the aortic wall after EVAR, whenever feasible, a complete endograft explantation was pursued. Surgical approaches included midline transperitoneal incision or thoracoabdominal approach. Depending on the inserted stent graft device and underlying pathology, an infrarenal or supra-celiac clamping site was established for hemorrhage control. Depending on the complication, aortic reconstruction was carried out using alloplastic (Dacron) or autologous (deep femoral vein/greater saphenous vein) as either ISR or EAR.

### Statistical data analysis

The data were collected in Excel (Microsoft, Redmond, Washington, USA), and statistical analysis was performed using SPSS Version 27 (IBM, Armonk, New York, USA). Data are presented (unless otherwise stated) according to scale levels as percentages or arithmetic means (M) and standard deviations (SD). Statistical analysis included Fisher’s exact test for categorical variables. Continuous variables were tested for normal distribution first and subsequently analyzed using the Mann-Whitney *U* test or *t*-test. A statistically significant result was assumed at a significance level of *p* ≤ 0.05. Survival curves were computed using Kaplan-Meier.

## Results

### Patient demographics and history

Fifty patients (88% men) were treated by OC after EVAR failure, and 31 patients had an elective OC and 19 patients an urgent procedure. Patients with EM-OC were older (ELE-OC: 71 ± 8 years vs. EM-OC: 78 ± 7 years). The relevant comorbidities and patient history are illustrated in Table 1.

**Table 1.** Patient demographics and indications for open conversion.

	Elective OC (*n* = 31)	Emergent OC (*n* = 19)	Total OC (*n* = 50)	*p*-Value
Age, years	71 ± 8	78 ± 7	74 ± 8	**0.001**
Men	26 (83.9%)	18 (94.7%)	44 (88.0%)	0.387

Comorbidities				
Arterial hypertension	24 (80.0%)	18 (94.7%)	42 (84.0%)	0.134
Hyperlipidemia	12 (38.7%)	8 (42.1%)	20 (40.0%)	1
Smoking	11 (35.5%)	3 (15.8%)	14 (28.0%)	0.197
DM	5 (16.7%)	3 (15.8%)	8 (16.0%)	1
CAD	12 (38.7%)	12 (63.2%)	24 (48.0%)	0.145
CHF	6 (19.4%)	6 (31.6%)	12 (24.0%)	0.496
AF	6 (19.4%)	8 (42.1%)	14 (28.0%)	0.110
COPD	7 (22.6%)	6 (31.6%)	13 (26.0%)	0.521
CKD	9 (29.0%)	6 (31.6%)	15 (30.0%)	1
ESRD	0 (0.0%)	1 (5.3%)	1 (2.0%)	0.380

Primary indication for EVAR				
AAA	30 (96.8%)	18 (94.7%)	48 (96.0%)	1
PAU	1 (3.2%)	1 (5.3%)	2 (4.0%)	1
				
Secondary interventions	12 (38.7%)	4 (21.1%)	16 (32.0%)	0.228
None	19 (61.3%)	15 (78.9%)	34 (68.0%)	0.228
Occlusion of feeding vessels	2 (6.5%)	2 (10.5%)	4 (8.0%)	0.629
Proximal graft extension	4 (12.9%)	0 (0.0%)	4 (8.0%)	0.248
Distal graft extension	3 (9.7%)	0 (0.0%)	3 (6.1%)	0.279
Crossover bypass implantation	3 (9.7%)	1 (5.3%)	4 (8.0%)	1
Mechanical recanalization	0 (0.0%)	1 (5.3%)	1 (2.0%)	0.380

Device				
Medtronic Endurant	18 (58.1%)	9 (47.4%)	27 (54.0%)	0.563
Cook Zenith	3 (9.7%)	4 (21.1%)	7 (14.0%)	0.404
Terumo Anaconda	4 (12.9%)	5 (26.3%)	9 (18.0%)	0.273
AFX/Endologix	3 (9.7%)	1 (5.3%)	4 (8.0%)	1
GORE	2 (6.5%)	0 (0.0%)	2 (4.0%)	0.519
Aorfix/Lombard	1 (3.2%)	0 (0.0%)	1 (2.0%)	1
				
Fixation site				
Suprarenal	24 (77.4%)	14 (73.7%)	38 (76.0%)	1
Infrarenal	7 (22.6%)	5 (26.3%)	12 (24.0%)	1
				
External implantation	28 (90.3%)	18 (94.7%)	46 (92.0%)	1
Lost to follow-up	5 (16.1%)	12 (63.2%)	17 (34.0%)	**0.002**

Indication for OC				
Stent-graft limb occlusion	6 (19.4%)	1 (5.3%)	7 (14.0%)	0.229
Endoleak	22 (71.0%)	15 (78.9%)	37 (74.0%)	0.742
Ia	9 (40.9%)	11 (73.3%)	20 (54.1%)	0.092
Ib	3 (13.6%)	3 (20.0%)	6 (16.2%)	0.670
II	7 (31.8%)	0 (0.0%)	7 (18.9%)	**0.028**
III	2 (9.1%)	1 (6.7%)	3 (8.1%)	1
V	1 (4.5%)	0 (0.0%)	1 (2.7%)	1
Rupture	0 (0.0%)	15 (78.9%)	15 (30.0%)	**0.001**
AGI	3 (9.7%)	3 (15.8%)	6 (12.0%)	0.661
				
Time implantation to OC, months	66 ± 49	66 ± 36	66 ± 44	0.648

Continuous data are presented as mean ± standard deviation and categorical dichotomous data as counts and percentages, unless otherwise stated.

OC: open conversion, DM: diabetes mellitus, CAD: coronary artery disease, CHF: congestive heart failure, AF: atrial fibrillation, COPD: chronic obstructive pulmonary disease, CKD: chronic kidney disease, ESRD: end-stage renal disease ASA: American Society of Anesthesiologists, EVAR: endovascular aortic repair, AAA: abdominal aortic aneurysm, PAU: penetrating aortic ulcer.

The most common primary indication for EVAR was infrarenal AAA in 48 patients (96%) and penetrating aortic ulcer in two patients (4%). Primary implantation was performed at an external institution in 92% (*n* = 46) of all cases. Following the primary intervention, a total of 17 patients (34%) did not participate in a regular follow-up program with higher rates in urgently treated patients (ELE-OC: 16% vs. EM-OC: 63%). Sixteen patients required secondary interventions after the initial stent graft implantation to treat EL type Ia, Ib, II, and chronic limb ischemia.

### Indications for OC

The most common indication for ELE-OC (see Table 1) included various types of persisting endoleaks in 22 patients (71%). In the case of EM-OC, the most common indication was an aortic rupture in 15 patients (79%) provoked by EL type Ia in 11 patients (73%), EL type Ib in three patients (20%), and EL type III in one patient (7%). Further indications included three patients (16%) treated urgently for septic or hemorrhagic shock because of fulminant AGI with consecutive aortointestinal fistula and one case (5%) of ALI because of stent graft limb occlusion.

Formerly inserted graft devices were produced by seven different manufacturers. In both subgroups, the most common fixation site of the stent graft was suprarenal (38 patients; 76%).

### Surgical treatment

Forty-two (84%) of all patients underwent transperitoneal midline incision, and in eight patients (16%), a thoracoabdominal approach was performed. The mean time of surgery was 289 ± 117 min. The characteristics of the surgical procedure are illustrated in Table 2.

**Table 2.** Characteristics of the surgical procedure.

	Elective OC (*n* = 31)	Emergent OC (*n* = 19)	Total OC (*n* = 50)	*p*-Value
Operative times, minutes	284 ± 114	298 ± 126	289 ± 117	0.690

Clamping location				
Infrarenal	9 (29.0%)	4 (21.1%)	13 (26.0%)	0.742
Supra-celiac	22 (71.0%)	15 (78.9%)	37 (74.0%)	0.742
Temporary supra-celiac	20 (64.5%)	10 (52.6%)	30 (60.0%)	0.533
Total supra-celiac	2 (6.5%)	5 (26.3%)	7 (14.0%)	0.089
				
Supra-celiac clamping time, minutes	10 ± 9	11 ± 8	11 ± 9	0.362
Temporary supra-celiac	7 ± 4	6 ± 2	7 ± 3	0.723
Total supra-celiac	34 ± 1	22 ± 3	25 ± 6	0.051

Completeness of graft explantation				
Total	25 (80.6%)	15 (78.9%)	40 (80.0%)	1
Partial	5 (16.1%)	2 (10.5%)	7 (14.0%)	1
Left in situ	1 (3.2%)	2 (10.5%)	3 (6.0%)	0.549
				
Extracorporeal membrane oxygenation	2 (6.5%)	0 (0.0%)	2 (4.0%)	0.519
Renal cold crystalloid perfusion	0 (0.0%)	5 (26.3%)	5 (10.0%)	0.005

Reconstruction procedure				
ISR	29 (93.5%)	17 (94.4%)	46 (93.9%)	1
EAR	2 (6.5%)	1 (5.6%)	3 (6.1%)	1

Reconstruction material				
Alloplastic	28 (90.3%)	15 (78.9%)	43 (86.0%)	0.404
Autologous	2 (6.5%)	2 (10.5%)	4 (8.0%)	0.629

Configuration of reconstruction				
Tube	6 (19.4%)	8 (42.1%)	14 (28.0%)	0.110
Aortobiiliac	17 (54.8%)	6 (31.6%)	23 (46.0%)	0.148
Aortobifemoral	3 (9.7%)	1 (5.3%)	4 (8.0%)	1
Aortomonoiliac/-monofemoral	2 (6.5%)	1 (5.3%)	3 (6.0%)	1
Proximal re-fixation	1 (3.2%)	1 (5.3%)	2 (4.0%)	1
Aortic stump ligation EAR	1 (3.2%)	1 (5.3%)	2 (4.0%)	1
Aortomonofemoral + CO-BP	1 (3.2%)	0 (0.0%)	1 (2.0%)	1

Continuous data are presented as mean ± standard deviation and categorical dichotomous data as counts and percentages, unless otherwise stated.

ISR: in situ repair, EAR: extra-anatomic repair, CO-BP: crossover-bypass.

Supra-celiac clamping was obtained in 37 (74%) of the patients. Thirty patients (60%) had temporary supra-celiac clamping for seven minutes (range 6–15 min), followed by infrarenal clamping after proximal detachment of the stent graft. Total supra-celiac clamping was necessary for seven patients (14%) for 25 min (range 17–34 min). In two electively performed cases (7%), total supra-celiac clamping was supported by extracorporeal membrane oxygenation devices to ensure visceral perfusion, whereas in five emergency cases (10%), we followed a “clamp and sew” approach supported by renal cold perfusion.

Reconstruction was performed as ISRs in 94%. In cases of extensive graft infection or unsuccessful recanalization of stent graft limb occlusion, EARs were performed using aortic stump ligation and axillobifemoral graft implantation in two patients (4%) and aorto-monofemoral graft implantation and subsequent crossover bypass implantation in one patient (2%).

### Outcomes

Postoperative complications can be divided into surgical and nonsurgical complications. Surgical complications included ischemic complications such as ALI in four patients which were immediately treated by transfemoral thrombectomy in all patients without any limb loss (see Table 3). Furthermore, ischemic colitis occurred in six patients resulting in Hartmann’s situation. Six patients (12%) required repeated surgical reintervention because of hemorrhagic complications, and seven patients (14%) suffered from surgical site infections, hence requiring surgical wound debridement and vacuum therapy. Further nonsurgical complications are illustrated in Table 3.

**Table 3.** Outcomes.

	Elective OC (*n* = 31)	Emergent OC (*n* = 19)	Total OC (*n* = 50)	*p*-Value
Complications				
Limb ischemia	3 (9.7%)	1 (5.3%)	4 (8.0%)	1
Ischemic colitis	2 (6.5%)	4 (21.1%)	6 (12.0%)	0.184
Renal failure	21 (67.7%)	14 (73.7%)	35 (70.0%)	0.757
Transient dialysis	9 (29.0%)	7 (36.8%)	16 (32.0%)	0.756
Persisting dialysis	0 (0.0%)	1 (5.3%)	1 (2.0%)	0.380
Hemorrhagic	3 (9.7%)	3 (15.8%)	6 (12.0%)	0.661
SSI	2 (6.5%)	5 (26.3%)	7 (14.0%)	0.089
Abdominal WHD	2 (6.5%)	4 (21.1%)	6 (12.0%)	0.404
Inguinal WHD	0 (0.0%)	1 (5.3%)	1 (2.0%)	0.380
MOF	9 (29.0%)	9 (47.4%)	18 (36.0%)	0.233
MI	0 (0.0%)	2 (10.5%)	2 (4.0%)	0.140
Pneumonia	7 (22.6%)	2 (10.5%)	9 (18.0%)	0.452
				
ICU LOS, days	12 ± 15	9 ± 7	11 ± 12	0.740
Hospital LOS, days	23 ± 19	19 ± 12	22 ± 17	0.889
Mortality				
In-hospital	1 (3.2%)	5 (26.3%)	6 (12.0%)	**0.024**

Continuous data are presented as mean ± standard deviation and categorical dichotomous data as counts and percentages, unless otherwise stated.

AKI: acute kidney injury, SSI: surgical site infections, WHD: wound healing disorder, MODS: multiorgan dysfunction syndrome, MI: myocardial infarction, ICU: intensive care unit, LOS: length of stay.

With six cases of in-hospital death, the 30-day in-hospital mortality rate was 12%, with higher rates in urgently treated patients (ELE-OC: 3% vs. EM-OC: 26%). One patient (2%) was transported to the operating theater under resuscitation for cardiopulmonary arrest because of hemorrhagic shock caused by aortic rupture; the patient died intraoperatively from persistent cardiac arrest after aortic clamping. Further reasons for 30-day in-hospital mortality were MOF because of septic shock caused by either colonic ischemia and bacterial translocation (EM-OC: *n* = 2) or bacterial translocation during surgery of low-grade aortic graft infection (ELE-OC: *n* = 1), MOF because of hemorrhagic shock caused by initial aortic rupture (EM-OC: *n* = 1), and therapy limitation based on the presumed will of the patient, as confirmed by the family after a complicated course of treatment (EM-OC: *n* = 1). Of these cases, one patient who was treated electively for low-grade aortic graft infection died because of multiorgan failure, with subsequent cardiac arrest because of systemic inflammatory response syndrome and septic shock on postoperative day one. Another patient returned to the theater four hours after the initial treatment of an aortic rupture after EVAR for presumed aortic bleeding. Intraoperatively, diffuse hemorrhagic was seen and treated with abdominal packing. Eight hours later, the patient died because of multiorgan failure, including disarranged blood coagulation induced by primary hemorrhagic shock. A further cause for secondary transportation to the theater was persisting septic shock, with CT-A morphological findings of bowel ischemia in two patients. According to the intraoperative findings and extent of bowel ischemia, surgical resection of the ischemic intestine was performed. In repeatedly performed second-look surgeries, the progression of bowel ischemia led to limited surgical options, and an interdisciplinary decision was made to limit therapy on postoperative days one and three, respectively. Another patient suffered from MOF, including the transient need for dialysis, postoperative ALI requiring surgical reintervention, hospital-acquired pneumonia with the need for prolonged intubation, and abdominal wound healing disorder. After frequent discussions with relatives, the decision for therapy limitation based on the presumed will of the patient, as confirmed by the family, was made, and the patient died on postoperative day five.

**Figure 1.**
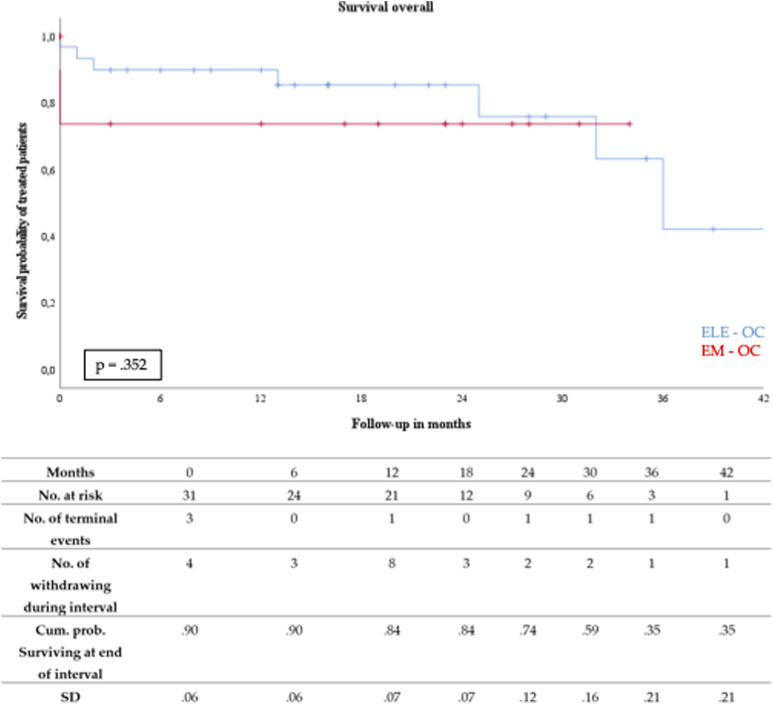
Kaplan–Meier estimated survival of elective- (ELE-OC) and emergency-open conversion (EM-OC) after EVAR.

The cumulative Kaplan–Meier estimate for survival (see Figure 1) revealed a one-year overall mortality rate of 12% (EL-OC: 10% vs. EM-OC: 26%). The mean time of follow-up was 16 months (range 0–48 months). Six patients (12%) died during the follow-up period. All were initially treated for elective OC.

The reasons for death during follow-up included two aortic-related deaths (4%): One patient was diagnosed with low-grade AGI of an aortobifemoral alloplastic graft 36 months after ELE-OC. Subsequently, graft explantation and aortic reconstruction using autologous material (deep femoral vein and greater saphenous vein) were performed, but the patient died on postoperative day five because of septic shock caused by bowel ischemia and bacterial translocation. One more patient died a month after EAR because of low-grade aortic graft infection with aortic stump ligation and axillobifemoral bypass implantation because of hemorrhagic shock caused by aortic stump blowout. A further cause of death during follow-up was one case of myocardial infarction two months after OC, and another two patients died from malignant diseases (colonic and prostatic carcinoma) 25 and 14 months after OC, respectively. Furthermore, one patient died 32 months after OC in December 2020 because of SARS-CoV-2 infection.

## Discussion

Although significant improvements in the expertise of vascular surgeons and medical technology have been made, an increase in OC after EVAR has been recorded in recent years.^31^ Despite this rarely occurring complication, with an incidence rate of 0.9%–5.3%,^32,33^ a further increasing incidence can be expected because of more frequently performed endovascular procedures, hence resulting in more complications.

Successful implantation of the endovascular stent graft includes complete elimination of the aortic aneurysm and a reduction in the risk of rupture and death. However, the most common indication for any secondary reintervention remains the persisting aneurysm perfusion.^7,8^ Accordingly, EL was by far the most common indication of OC. Particularly in cases of simultaneous aortic rupture, EL type I represents the most frequent indication for OC, here comparable to previous studies showing incidence rates of 52.0%^34^ to 82.4%.^31^

It has not been conclusively clarified which pathophysiological changes induce EL after EVAR. However, recent studies have shown increasing evidence of aortic wall destabilization induced by endovascular stent graft implantation. The combination of cellular apoptosis, inflammation, degradation of extracellular matrix proteins, and changes in cellular functions may result in a weakened aortic wall structure.^25,27,28^ In a recently published study, we have already provided evidence for EVAR-induced aortic wall destabilization. The ability of the aortic wall to respond to persisting or newly occurring luminal blood flow in the case of EL may be decreased because of impaired contractility in vascular smooth muscle cells caused by a decreased concentration of actin, tropomyosin, and troponin. Furthermore, the continuing degradation of extracellular matrix proteins, such as collagen, might support this structural instability.^28^

As long as EVAR is performed successfully without persisting perfusion of the aneurysm, these structural alteration problems will be limited and not cause significant trouble. However, this can change if EL occurs. Particularly in the case of EL types I and III, in which high-flow blood pressure leads to increased wall stress, the weakened aortic wall may not withstand, resulting in fatal aortic rupture and urgent OC.

Mortality rates after OC vary in the literature from 0.0%^32^ up to >40.0%^35,36^ in earlier series. More recently published studies reported 30-day mortality rates of around 10.0%–23.8%.^33,37–39^ The mortality rates in our study are similar. Although some authors such as Brinster et al.^32^ have reported mortality rates of 0.0%, we assume that we were more frequently dealing with a high-risk patient cohort combined with a high number of emergency surgeries and challenging suprarenal localizations. Because OC after EVAR is a high-risk procedure itself, detailed preoperative preparation is often not feasible in an emergency, adding another challenge for anesthesiologists and surgeons. Additionally, we were frequently confronted with a disease associated with high mortality rates, even without a former inserted stent graft: the aortic rupture. In comparison, Brinster et al. reported only a single case of aortic rupture and a lower rate of emergency surgeries. In a study by Joo et al.^33^ mortality rates differed significantly between the elective and emergency surgery groups (4.1% vs. 33.3%). The mortality rate in our patient cohort for EM-OC was significantly higher than ELE-OC and included a high rate of patients initially presenting with aortic rupture after EVAR. In the largest study up to date, Scali et al.^40^ confirmed higher mortality rates in an emergency setting; they revealed a 37.0% mortality rate in the case of emergency surgery of 118 patients treated after EVAR. Even with nonelective primary OAR, the mortality rate was significantly higher (37.0% vs. 24.0%), and OC after EVAR was identified as an independent predictor of 30-day mortality, especially with underlying rupture and upper localization of aortic cross-clamping.

To prevent urgent OC because of aortic rupture caused by EL, the need for sufficient clinical and image morphological follow-ups should be emphasized.^41^ If follow-ups are insufficient or absent, the risk of late detection of complications or no detection at all increases, as does the risk of emergency surgery. Because we observed a significantly higher rate of patients formerly lost to follow-up ending in emergency surgery, early detection of complications, particularly high-flow EL, may prevent emergency OC by exhausting elective endovascular options. It will be of great interest to examine whether the extent of pathophysiological changes within the aortic wall progresses over time with the increasing duration of the inserted stent graft. This should be further investigated in experimental studies and would emphasize the need for sufficient follow-up and urgent treatment in the case of high-flow EL.

## Limitations

The current study is subject to different limitations. The most notable is the retrospective single-center study design and small sample size because of the rarity of this surgical procedure. The high rate of external implantations makes it impossible to collect all information on the primary implantation process, such as device characteristics or even if the implantation was made per the manufacturer’s instructions for use. Furthermore, the exact technique and quantity of former reinterventions could not be evaluated because most patients were transferred in an urgent situation from their primary treating clinics with a lack of accompanying information.

## Conclusion

Primary implantation of endovascular stent grafts in a well-selected patient cohort remains the standard of care for treating abdominal aortic aneurysm. Although the incidence rate of complications leading to OC after EVAR is low, we are dealing with a high-risk procedure that poses a considerable challenge to the interdisciplinary team, for example, vascular surgeons, anesthesiologists, and intensive care physicians.

In times of rising frequency of endovascular surgeries, a further increase can be expected. OC after EVAR is associated with high mortality rates. Particularly in cases of insufficient or absent follow-up, complications such as EL paired with a weakened aortic wall induced by the former stent-graft implantation pose an enormous risk for fatal aortic rupture, emergency OC, and subsequent higher mortality. A thorough understanding of the underlying disease and ongoing pathophysiological alterations in the aortic wall after EVAR is essential to prevent complications leading to OC. Therefore, besides recommending careful follow-up, we would like to encourage further research into the underlying pathophysiological processes leading to OC after EVAR.

## Abbreviations

AAA abdominal aortic aneurysm

AGI aortic graft infection

AKI acute kidney injury

ALI acute limb ischemia

CT-A computed tomography angiography

EAR extra-anatomic reconstruction

EL endoleak

ELE-OC elective open conversion

EM-OC emergency open conversion

EVAR endovascular aortic repair

ISR in situ reconstruction

MOF multiorgan failure

OAR open aortic repair

OC open conversion

SSI surgical site infection
